# Repeated recurrence after endovascular treatment for cerebral aneurysms: predictive clinical factors and optimal therapeutic management

**DOI:** 10.1007/s10143-025-03758-w

**Published:** 2025-08-16

**Authors:** Kota Kurisu, Hiroyuki Sakata, Yasushi Matsumoto, Atsushi Kanoke, Shunsuke Omodaka, Miki Fujimura, Hidenori Endo

**Affiliations:** 1 Department of Neuroendovascular Therapy, Kohnan Hospital, Sendai, Miyagi Japan; 2 Department of Neurosurgery, Hokkaido University Graduate School of Medicine, Sapporo, Hokkaido Japan; 3 Department of Neurosurgery, Kohnan Hospital, Sendai, Miyagi Japan; 4 Division of Development and Discovery of Interventional Therapy, Tohoku University Hospital, Sendai, Miyagi Japan; 5 Department of Neurosurgery, Tohoku University Graduate School of Medicine, Sendai, Miyagi Japan

**Keywords:** Cerebral aneurysm, Endovascular treatment, Coiling, Recurrence

## Abstract

Despite significant advances in endovascular treatment (EVT) of cerebral aneurysms, post-treatment recurrence necessitating multiple retreatments remains a severe concern. Herein, we investigated the clinical characteristics of aneurysms necessitating multiple retreatments after EVT (refractory aneurysms: R-ANs) and explored appropriate management strategies. This retrospective cohort study enrolled 1,045 aneurysms initially treated with EVT between 2016 and 2022. R-ANs were defined as cases requiring two or more retreatment sessions due to repeated recurrence or regrowth. Clinical data were retrospectively reviewed, and predictive factors were identified. Furthermore, the treatments performed and clinical outcomes were investigated. Of 1,045 aneurysms, 101 (9.7%) exhibited recurrence or regrowth requiring retreatment. Among these, 23 aneurysms (2.2%) requiring two or more retreatment sessions, which were classified as R-ANs. As for the predictive factors of R-AN development, large size (> 10 mm), and circumferential aneurysmal wall enhancement on vessel wall imaging (VWI) were identified as relevant and independent predictive factors, with odds ratios of 3.92 (95% CI, 1.36– 11.62), 8.02 (95% CI, 2.02– 53.6), respectively. In terms of therapeutic outcomes, repeat EVT sessions provided favorable long-term clinical outcomes (aneurysm stabilization, 85.0%; favorable occlusion, 65.0%) and low periprocedural complication rates (2.6%). This study identified the relevant predictive factors for R-AN development, suggesting that careful follow-up is essential in patients with these factors. Furthermore, repeated EVT sessions appears to be a safe and efficient treatment strategy for such challenging cases.

## Introduction

Rupture of cerebral aneurysms (CA) leads to aneurysmal subarachnoid hemorrhage (aSAH), a subtype of severe stroke associated with disability and mortality [1]. For patients with either ruptured or unruptured CAs, microsurgical clipping or endovascular treatment (EVT) are currently the available treatment options to prevent future or acute re-rupture [2].

Despite the efficacy of microsurgical clipping in completely occluded aneurysms, the less invasive nature of EVT and its associated shorter recovery times make it the preferred option. Several nationwide cohort studies [3, 4] have reinforced EVT as the preferred option in many developed countries. This is supported by the large clinical trials, International Subarachnoid Aneurysm Trial (ISAT) [5, 6], which demonstrated superior short- and mid-term clinical outcomes with EVT, compared to microsurgical clipping, particularly for ruptured CAs. Further, the Barrow Ruptured Aneurysm Trial (BRAT) [7] also showed favorable long-term outcomes in patients who underwent EVT. In addition, recent advances in endovascular techniques and devices reinforce this trend and expanding the indications of EVT. On the other hand, even in this advanced era of EVT, the long-term durability of EVT remains as unresolved concern [8, 9].

Previous reports have demonstrated that recurrence and regrowth after EVT occur in approximately 10–20% of aneurysms, even those treated with modern neck bridging devices [10]. For these recurrent CAs, optimal management strategy, whether and when additional retreatment should be performed, and what is the appropriate method, and so on, are still unclear due to the paucity of large clinical trials and heterogeneity of patient conditions [9, 11]. Retreatment strategies for such challenging cases are decided on a case-by-case basis in actual clinical settings, considering the location and morphology of aneurysm, the type of recurrence, background, preferences, and various other factors [9, 11]. Moreover, we also encounter the cases showing multiple recurrences and regrowth despite repeated EVT sessions, which are more complex and challenging to treat [11]. However, little is known about the clinical characteristics and optimal management strategy for these challenging CAs, referred to herein as ‘refractory aneurysms (R-ANs)’, owing to a lack of available clinical data.

Here-in, we retrospectively reviewed our clinical experiences to elucidate the clinical characteristics of ANs which showing such refractory clinical courses, and to elucidate appropriate management strategies. Findings from this study would help to better understand this rare but challenging condition and will encourage discussion on the optimal management strategies.

## Material and methods

### Study design

This study was approved by the Kohnan Hospital Institutional Review Board (approval number: 2023–0118-03). As this retrospective observational study has a very low risk to the study participants, an opt-out arrangement was applied, by providing the necessary information to make an informed decision and include safeguards to protect privacy. We retrospectively analyzed 1,087 consecutive patients with 1,145 aneurysms initially treated with EVT between January 2016 and December 2022. Clinical data for R-ANs were extracted from patients’ medical records. In this study, R-ANs were defined as aneurysms that showed repeated recurrence and regrowth during the postprocedural follow-up period, requiring at least two additional treatment sessions (three treatment sessions in total). Planned multistage endovascular treatment, such as partial aneurysm coil embolization in the acute phase of a ruptured aneurysm, followed by radical coil embolization or flow diverter (FD) treatment in the chronic phase, was considered a single treatment session. Patients with dissecting aneurysms were excluded. To elucidate the clinical characteristics of R-ANs, a logistic regression analysis was conducted between R-ANs and control aneurysms, including aneurysms with no evidence of recurrence, regrowth, or additional retreatment during at least 3 years of observation.

### Patient demographics and aneurysm characteristics

Patient demographics and aneurysm characteristics were retrospectively extracted from medical records. The following patient data were collected: sex, age, comorbidities, previous diseases, and clinical presentation of the aneurysm (unruptured/ruptured, evolving, symptomatic, or asymptomatic). An evolving aneurysm was defined as one that demonstrated either radiographic enlargement or morphological alterations, such as daughter sac formation [12, 13]. In addition, aneurysms that presented with, or showed worsening of, aneurysm-related neurological symptoms—such as oculomotor nerve palsy or visual disturbances—were also classified as evolving aneurysms [14]. Aneurysm characteristics, including shape, maximum diameter, dome-to-neck (D/N) ratio, and location (anterior/posterior circulation, side-wall type/end-wall type) were assessed using 3D digital subtraction angiography (DSA). Given that aneurysmal wall enhancement with Gadolinium on T1-weighted vessel wall imaging (VWI) has been reported as an important clinical predictor of aneurysm instability [8, 15], the VWI status (no enhancement/focal enhancement/circumferential wall enhancement) [12, 16] was also assessed at initial EVT.

### Endovascular treatment procedures

Endovascular treatment (EVT) was performed by experienced operators under either general anesthesia or deep sedation with local anesthesia, using femoral or radial approaches. Bare platinum coils (Axium Prime [Medtronic], Target [Stryker], and ED [Kaneka Medix] coils, Optima [Balt]) and bioactive coils (HydroCoil, MicroVention Terumo) were used for coil embolization. For unruptured cerebral aneurysms, stent-assisted coil embolization was selected if required, depending on the morphology of the aneurysm, using LVIS (MicroVention Terumo) or Neuroform (Stryker) stents, and PulseRider (Cerenovus, Johnson, & Johnson) for neck reconstruction. Furthermore, flow diversion treatment, particularly for large, wide-neck, sidewall-type aneurysms, was selected if required, using Pipeline [Medtronic] and FRED [MicroVention Terumo]. Embolization status was qualitatively assessed immediately after and at follow-up cerebral angiography, according to the Raymond Roy (RR) classification scheme [17]. Briefly, RR class 1 indicates complete occlusion, class 2 indicates residual neck, and class 3 indicates residual aneurysm.

Patients with unruptured aneurysms received dual antiplatelet therapy (100 mg aspirin and 75 mg clopidogrel) at least 2 weeks prior to the procedure. Patients with ruptured aneurysms did not receive antiplatelet medications before the procedure. Antiplatelet therapy was discontinued within three months in patients treated with coil embolization alone. For patients who underwent stent-assisted coil embolization or flow diversion treatment, dual antiplatelet therapy was maintained for 6 months, and subsequently switched to monotherapy for 6 months, based on follow-up angiographic findings.

### Post-procedural follow-up and retreatment protocol

All patients underwent follow-up with MR and cerebral angiography at 6, 12, and 24 months after treatment, and annually thereafter. In cases where aneurysm recurrence or regrowth was suspected on annual MR, repeat angiography was performed. At each imaging time point, aneurysm occlusion status was assessed using the Raymond-Roy classification [18]. Aneurysms were considered to have achieved stabilization if there was no evidence of progressive enlargement of residual components or worsening of body filling across at least two consecutive follow-up imaging assessments, and no further retreatment was required. The therapeutic strategy, including simple coil embolization, stent-assisted coil embolization, flow diversion treatment, or a combination of EVT and direct surgery, was decided on a case-by-case basis, considering factors such as patient background, aneurysm location, and angiographical morphology.

### Statistical analysis

All data were analyzed using standard descriptive statistics. Continuous variables are expressed as the mean ± standard deviation (SD) or median and interquartile range (IQR). For categorical variables, the number of participants and percentages were calculated. Fisher’s exact test or the χ2 test were used to compare categorical variables, while the Mann–Whitney U test (for non-normally distributed data) or unpaired Student’s t-test (for normally distributed data) were used for continuous variables. Univariate logistic regression analysis was applied to obtain odds ratios (ORs) and 95% confidence intervals (CIs) for observed events of interest. Multivariate logistic regression analysis was also performed to identify the most relevant predictive factor. Considering the limited number of events (*n* = 23 for R-ANs), each multivariate model was restricted to a maximum of two independent variables to reduce the risk of statistical overfitting and to ensure the reliability of the estimates. Statistical significance was set at *p* < 0.05. Statistical analyses were performed using the GraphPad Prism software (version 10.0.2; San Diego, CA, USA).

## Results

### Study population

**Fig. 1:**
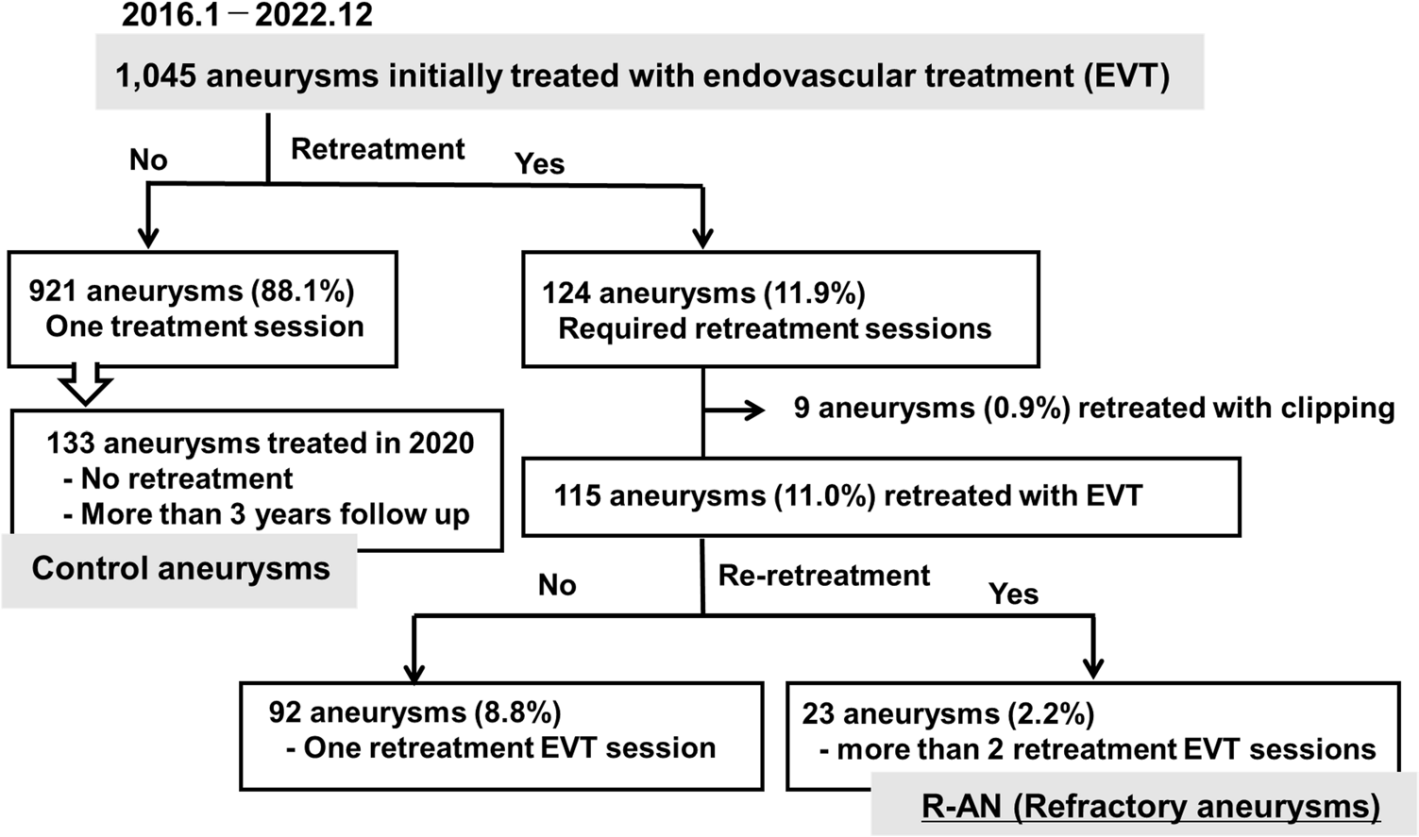
Study population of the present study. Study population enrolled in the present study. Of the 1,045 aneurysms, 124 aneurysms (11.9%) required additional retreatment for recurrence of aneurysms. Of these, 23 (2.2%) required more than one EVT re-retreatment session for repeat recurrence, which were dichotomized into an R-AN group. Of the 921 aneurysms that showed no evidence of recurrence or regrowth after initial EVT, 133 treated in 2020 could have at least three years of follow-up period, were used as the control group

The study population is shown in Fig. 1. Of the 1,045 aneurysms initially treated with EVT, 921 (88.1%) required only one EVT session without any recurrence, and 124 (11.9%) required additional retreatment for aneurysm recurrence. Of these, 115 cases (11.0%) were managed with further EVT, while 9 cases were managed with microsurgical clipping. For these 9 cases, none of the patients experienced periprocedural complications or subsequent recurrence during follow-up period.

Among 115 aneurysms (11.0%) retreated with additional EVT sessions, 23 aneurysms (2.2%) required more than one re-retreatment EVT session for repeat recurrence and were classified into the R-AN group. Importantly, no patients in the R-AN group underwent direct surgical clipping after multiple EVT sessions.

For comparative analysis, a control group was selected from the 921 aneurysms that showed no evidence of recurrence or regrowth after initial EVT. Specifically, 133 aneurysms treated in 2020 and followed for at least 3 years were used as the control group for further analysis.

### Clinical characteristics

The clinical characteristics of the R-AN and control groups are summarized in Table 1. The R-AN group had a significantly higher prevalence of hyperlipidemia than the control group (*p* = 0.035; 52.2% vs. 29.3%). And although not significant, we also observed a trend towards a higher prevalence of hypertension in the R-AN group (*p* = 0.137, 69.6% vs. 52.6%). In terms of clinical presentation, the rate of ruptured aneurysms was significantly higher in the R-AN group than in the control group (*p* = 0.003, 52.2% vs. 21.1%). Further, although the difference was not statistically significant, the rate of evolving aneurysms was higher in the R-AN group than in the control group (*p* = 0.157; 43.5% vs. 28.6%). Regarding aneurysmal characteristics, aneurysms in the R-AN group were significantly larger than those in the control group (*p* = 0.003, 12.0 ± 5.9 mm vs. 8.2 ± 5.1 mm), with a significantly higher proportion of large (≥ 10 mm) aneurysms (*p* < 0.001, 56.5% vs. 22.6%). VWI analysis showed that the RCA group had a significantly higher rate of aneurysmal wall enhancement (*p* = 0.001, 90.5% vs. 42.7%), particularly circumferential wall enhancement (*p* < 0.001, 66.6% vs. 27.4%) than controls. There were no significant differences in aneurysm location, shape, D/N ratio, or intra-aneurysmal thrombosis.

**Table 1:** Chrinical characteristics of refractory aneurysms

	R-AN (*n* = 23)	Control (*n* = 133)	*p*-value
Backgrounds			
Age, yrs	62.9 ± 12.9	64.1 ± 13.7	0.403
Female (%)	19 (82.6)	99 (74.4)	0.684
Comorbidities			
Hypertension	16 (69.6)	70 (52.6)	0.137
Dyslipidemia	12 (52.2)	39 (29.3)	*0.035*
Diabetes Miletus	2 (8.7)	16 (12.0)	0.646
Clinical presentations			
Ruptured aneurysm	12 (52.2)	28 (21.1)	*0.003*
Symptomatic aneurysm	3 (13.0)	24 (18.0)	0.873
Evolving aneurysm	10 (43.5)	38 (28.6)	0.157
Multiple aneurysm	9 (39.1)	51 (38.3)	0.943
Aneurysm characteristics			
Anterior Circulation	13 (65.2)	99 (74.4)	0.360
End-wall type	15 (65.2)	75 (56.8)	0.431
Irregular shape	8 (34.8)	45 (34.1)	0.949
Aneurysmal parameters			
Maximum diameter (mm)	12.0 ± 5.9	8.2 ± 5.1	*0.003*
Large (> 10 mm)	13 (56.5)	30 (22.6)	< *0.001*
Dome/Neck ratio	1.6 ± 0.6	1.6 ± 0.5	0.808
Vessel wall Imaging			
Aneurysmal wall enhancement	19 (90.5)	50 (42.7)	*0.001*
Circumferential enhancement	14 (66.6)	32 (27.4)	< *0.001*
Focal enhancement	5 (23.8)	18 (15.4)	0.751
Thrombosis	2 (8.7)	3 (2.3)	0.657
Treatment modality for initial EVT			
Coil embolization	10 (43.5)	58 (43.6)	> 0.999
Stent assisted coil embolization	12 (52.2)	54 (40.6)	0.363
Flow diverter	1 (4.3)	21 (15.7)	0.202

Values are shown as number (%) or mean ± SD; *R-AN* Refractory aneurysms, *yrs* years, *EVT* endovascular treatment

### Predictive factors for R-AN

**Fig. 2:**
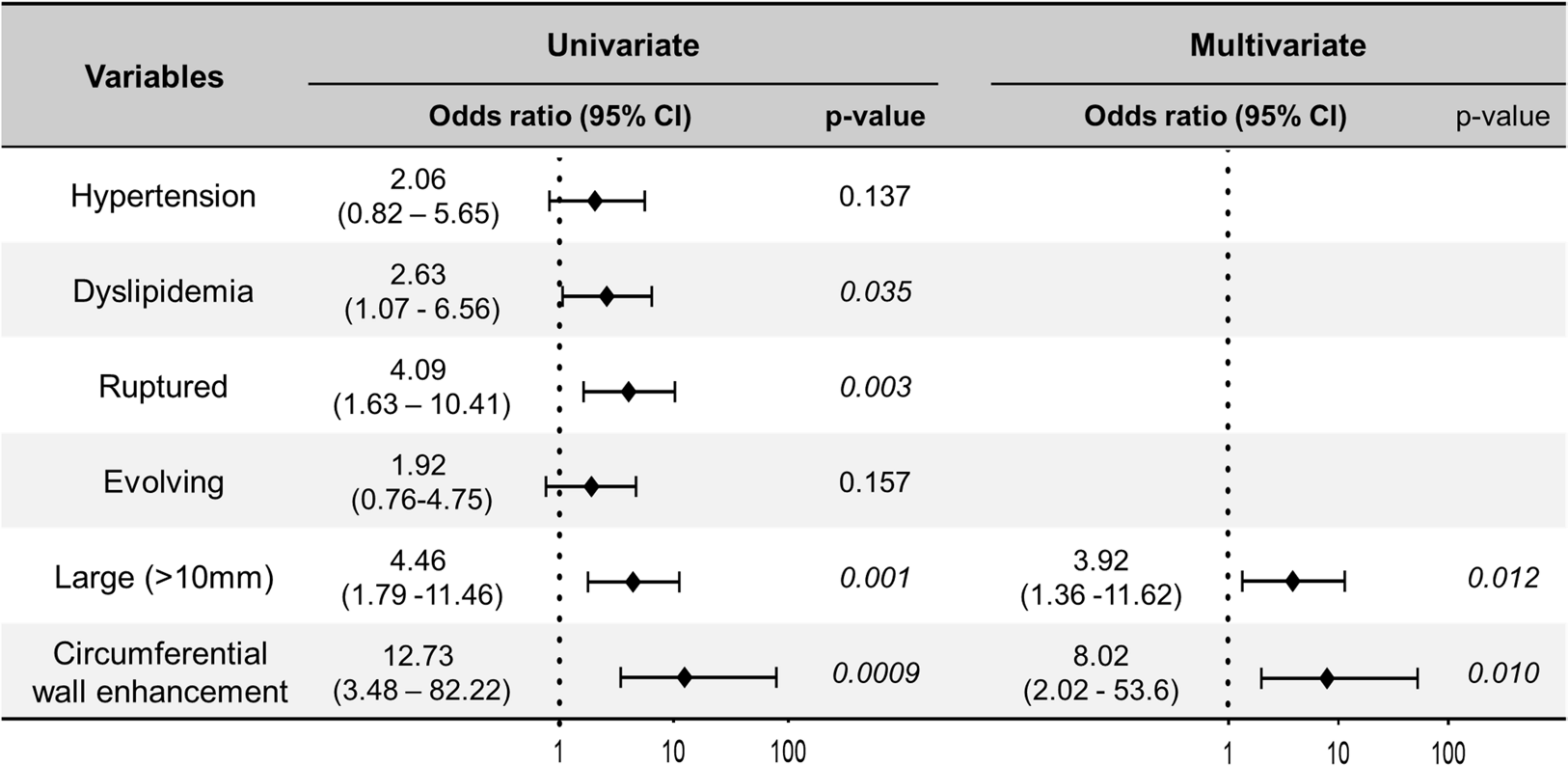
Clinical predictors for refractory aneurysms (R-ANs) development. Univariate logistic regression identified dyslipidemia, ruptured status, large aneurysm size (> 10 mm), and circumferential aneurysmal wall enhancement on vessel wall imaging (VWI) as significant predictors for R-AN development. Multivariate logistic regression, limited to two variables, demonstrated that large aneurysm size and circumferential wall enhancement were independent predictive factors for R-ANs. The odds ratio (OR) for large size was 3.96 (95% CI, 1.36–11.62), and for circumferential wall enhancement was 8.02 (95% CI, 2.02–53.60)

Univariate logistic regression analysis of clinical and radiographic variables revealed that dyslipidemia (odds ratio [OR], 2.63; 95% confidence interval [CI], 1.07–6.56), ruptured status (OR, 4.09; 95% CI, 1.63–10.41), large aneurysm size > 10 mm (OR, 4.96; 95% CI, 1.79–11.46), and circumferential aneurysmal wall enhancement on VWI (OR, 12.73; 95% CI, 3.48–82.22) were significantly associated with the development of R-ANs (Fig. 2). Among these, the two variables with the lowest p-values—large aneurysm size and circumferential wall enhancement—were selected for inclusion in the multivariate logistic regression model to minimize overfitting due to the limited number of events. Multivariate analysis confirmed that both variables were independent predictive factors for R-AN development, with ORs of 3.96 (95% CI, 1.36–11.62) and 8.02 (95% CI, 2.02–53.6), respectively, indicating their relevance as risk factors for this R-AN development.

### Initial endovascular treatment for R-AN

**Fig. 3:**
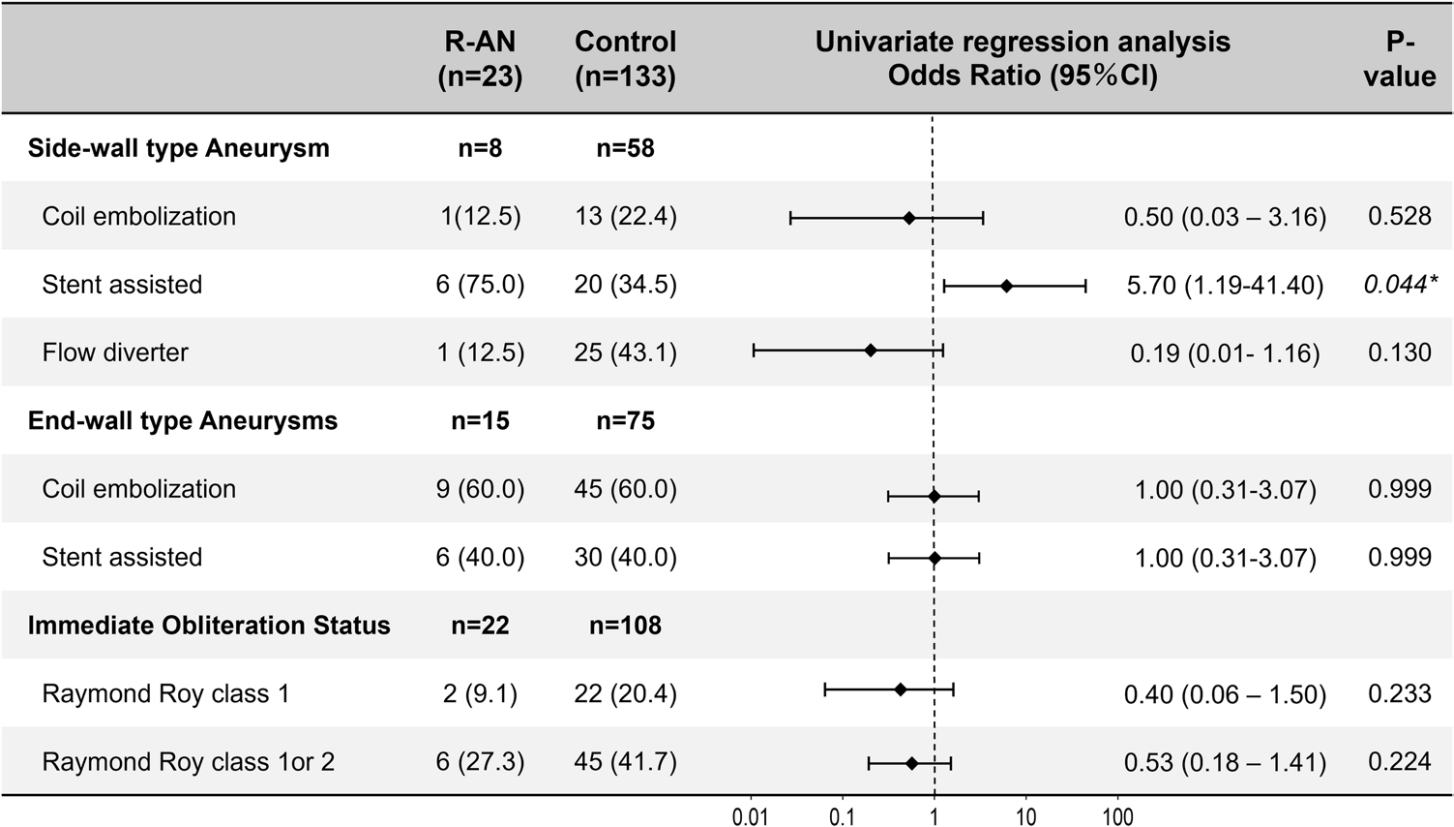
Initial treatment effect on development of refractory aneurysms (R-ANs). There was no influence of selected treatments in initial endovascular treatment session on R-AN development. However, we observed a trend that fewer R-AN cases were more likely to achieve Raymond Roy (RR) class 1 or 2 obliteration status (class 1, 9.1% vs. 20.4%, *p* = 0.233; class 1 or 2, 27.3% vs. 41.7%, *p* = 0.224), with odds ratios of 0.40 (95% CI, 0.06–1.50) and 0.53 (95% CI, 0.18–1.41) for RR class 1 and RR class 1 or 2, respectively

To investigate the effect and outcome of selected initial EVT strategy, univariate regression analysis was performed to allow comparison with the control group (Fig. 3). As for the treatment for side-wall type aneurysms (R-AN group, 8 cases, 34.8%; control group, 58 cases, 43.6%), the bias toward stent-assisted coil embolization was significantly higher in the R-ANs than the control group (*p* = 0.044, 75.0% vs. 34.5%), with an odds ratio of 5.70 (95% CI, 1.19–41.40). This bias may be explained by the selection bias in favor of stent-assisted coil embolization for morphologically challenging aneurysms. Furthermore, although did not reach statistically significance, fewer cases in the R-AN group were receiving FD treatment (*p* = 0.13, 12.5% vs. 43.1%), with an odds ratio of 0.19 (95% CI, 0.01 - 1.16). This could be explained high efficiency of FD treatment in terms of aneurysm occlusion. Regarding the treatment for end-wall type aneurysms (R-AN group, 15 cases, 65.2%; control group, 75 cases, 56.4%), there were no significant difference in the distribution of initial treatment selection (coil embolization/stent-assisted coil embolization).

The effect of embolization status following the initial EVT was assessed in both R-AN and control group, excluding cases treated with FD (Fig. 3). Although differences did not reach statistical significance, there was a clear trend indicating that a more favorable occlusion status was associated with a lower likelihood of R-ANs development. Specifically, the odds ratios for developing R-ANs were 0.40 (95% CI, 0.06–1.50) for Raymod-Roy class 1 and 0.53 (95% CI, 0.18–1.41) for Raymod-Roy Class 1 or 2 occlusion. These findings suggest that the degree of aneurysm occlusion achieved at the initial EVT may be an important factor influencing the risk of R-AN development.

### Summary of the repeated retreatments for R-AN

A total of 78 procedural sessions were performed for 23 R-ANs (side-wall type, 24 sessions for 8 aneurysms; end-wall type, 54 sessions for 15 aneurysms), with a median of 3 [3, 4] sessions per aneurysm. The procedure details are described in Table 2, including 35 (44.9%) sessions of coil embolization, 35 (44.9%) sessions of stent-assisted coil embolization, five (6.4%) sessions of flow diverter (FD) treatment, one (1.3%) session of parent artery occlusion, and two (2.6%) sessions of combined treatment with direct surgery.

**Table 2:** Summary of entire treatments for refractory aneurysms

	R-AN	Side-wall type	End-wall type
Number of ANs	23	8	15
Number of procedural sessions	78	24	54
Number of procedural sessions per AN	3 [3-4]	3 [3-3]	3 [3-4]
Performed Procedures (%, per sessions)			
Coil embolization	35 (44.9)	7 (9.0)	28 (35.9)
Stent assisted coil embolization	35 (44.9)	13 (16.7)	22 (28.2)
single stent use	28 (35.9)	13 (16.7)	15 (19.2)
pulse-rider use	3 (3.8)	0 (0.0)	3 (3.8)
multiple stent uuse	4 (5.1)	0 (0.0)	4 (5.1)
Flow diverter treatment	5 (6.4)	3 (3.8)	2 (2.6)
Parent artery occlusion	1 (1.3)	1 (1.3)	0 (0.0)
Combination of direct surgery	2 (2.6)	0 (0.0)	2 (2.6)
Clinical oucomes (%, per treated aneurysms)			
Follow up period, months	36 [25-49]	28 [25-41.5]	39 [26.5-70]
Latest Aneurysmal outcomes			
Raymond Roy class 1	9 (45.0)	4(20.0)	5 (25.0)
Raymond Roy class 1 or 2	13 (65.0)	4 (20.0)	9 (45.0)
Aneurysm stabilization	17 (85.0)	6 (30.0)	11 (55.0)
Neurological worsening event during follow up period	0 (0.0)	0 (0.0)	0 (0.0)
Latest functional outcome (mRS)	0 [0-1.5]	0 [0-2.3]	0 [0-0.5]
Procedure related complications (%, per sessions)	3 (3.8)	2 (2.6)	1 (1.3)
Ischemic complication	3 (3.8)	2 (2.6)	1 (1.3)
Hemorrhagic complication	1 (1.3)	0 (0.0)	1 (1.3)
mRS worsening complication	2 (2.6)	1 (1.3)	1 (1.3)

Values are shown as number (%) or mean ± SD or median [interquartile range]; *R-AN* refractory aneurysm, *AN* aneurysm, *mRS* modified rankin scale

**Fig. 4:**
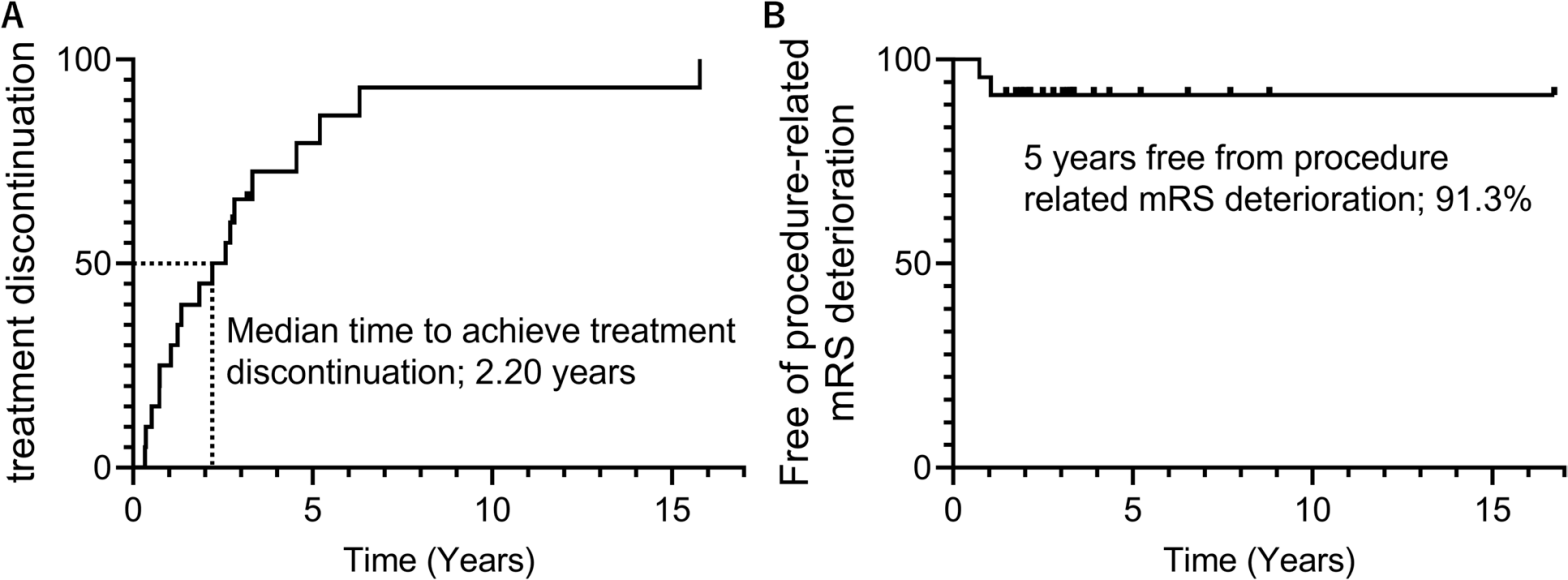
Clinical outcomes of multiple endovascular treatment (EVT) sessions for refractory aneurysms (R-ANs). (**A**) Kaplan–Meier analysisshowing that a favorable clinical outcome could be achieved by with repeated retreatment sessions for R-ANs, and the median time required to discontinue treatment for R-ANs was 2.20 years. (**B**) Procedure related modified rankin scale (mRS) deterioration was low, and the 5 years free from procedure related mRS deterioration was 91.3%

Long-term outcomes were assessed in 20/23 patients, with a median follow-up of 36 [25–49] months. At the last follow-up angiography, the aneurysm occlusion status was classified as RR class 1 in nine (45%) cases and RR class 1 or 2 in 13 (65%) cases. Overall, aneurysm stabilization was achieved in 17 (85.0%) of the 20 R-ANs for which repeat retreatment was discontinued as post-procedural follow-up angiography confirmed no further evidence of recurrence or regrowth. The Kaplan–Meier analysis (Fig. 4A) showed that a favorable clinical outcome could be achieved with repeated retreatment sessions for R-ANs, and that the median time required to discontinue treatment for R-ANs was 2.20 years.

Procedure-related complications and ischemic complications occurred in 3 (3.8%) sessions each; one of the ischemic cases further developed a hemorrhagic transformation in the ischemic lesion. Procedure-related worsening of the mRS was observed in two (2.6%) sessions, indicating an acceptably low incidence of serious complications associated with repeated treatment for R-ANs. Kaplan–Meier analysis (Fig. 4B) revealed a low incidence of procedure-related mRS deterioration caused by repeat retreatment sessions for R-ANs, and 91.3% of cases were kept free of procedure-related mRS deterioration over 5 years.

### Illustrative case

**Fig. 5:**
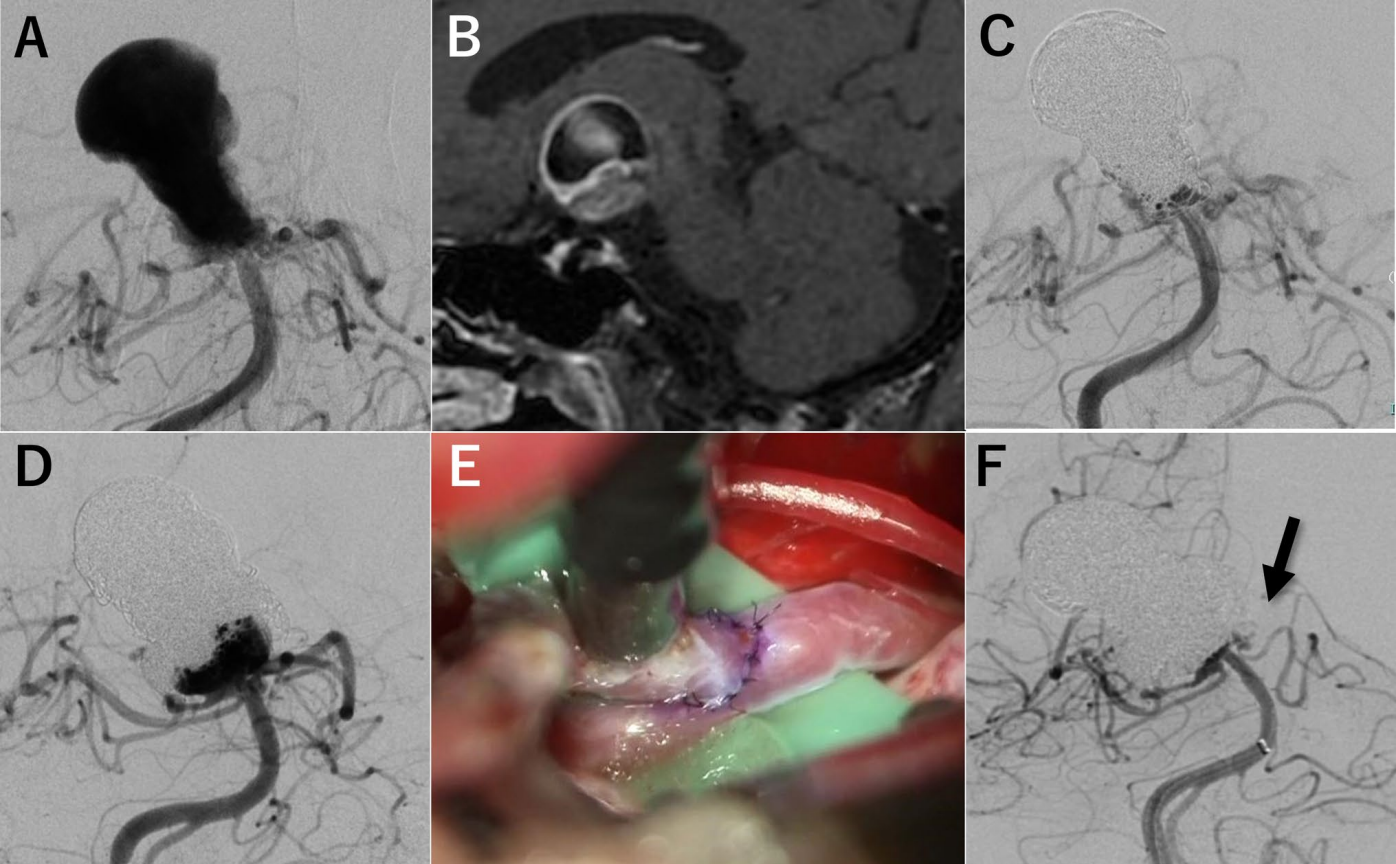
Illustrative case of a 62-year-old female with a refractory aneurysm. (**A**) Initial cerebral angiography revealed a large BA aneurysm with a maximum diameter of 28 mm. (**B**) Vessel wall imaging (VWI) showed circumferential aneurysmal wall enhancement. (**C**) Initial endovascular treatment (EVT) with simple coil embolization resulted in favorable aneurysm obliteration. (**D**) After three retreatment sessions with EVT, a fourth recurrence was confirmed. (**E**) For combined treatment of direct surgery and EVT, STA-PCA bypass was performed followed by clipping of the origin of the left PCA. (**F**) Angiography following additional coil embolization showing favorable aneurysm obliteration (Raymond Roy class 2) and disappearance of antegrade flow in the PCA (arrow)

A 62-year-old woman presented with progressively worsening visual field defects. Cerebral angiography (Fig. 5A) revealed a large (maximum diameter, 28 mm) basilar artery (BA) aneurysm with circumferential aneurysmal wall enhancement on VWI (Fig. 5B). Initial EVT was performed using simple coil embolization, achieving favorable obliteration of the aneurysm (Fig. 5C). However, the patient experienced repeated recurrence and regrowth, requiring three additional EVTs. A fourth recurrence was confirmed 22 months after the initial treatment (Fig. 5D), and a fourth retreatment session with direct surgery followed by EVT was planned. Using the left subtemporal approach, Superficial Temporal Artery -Posterior Cerebral Artery (PCA) bypass (Fig. 5E) and clipping of the left PCA origin were performed to achieve flow modulation of the BA and the arising aneurysm, followed by an intra-aneurysmal coil embolization. The postprocedural angiogram (Fig. 5F) showed favorable aneurysm obliteration (RR class 2), with the disappearance of the antegrade flow of the left PCA (arrow). The patient's postprocedural course was uneventful, and she was followed up with an mRS score of 1, without evidence of aneurysm recurrence.

## Discussion

The present study focused on R-ANs that exhibited multiple recurrences requiring repeated retreatment sessions, and is the first to reveal their detailed clinical characteristics. We demonstrated that large size (> 10 mm), and circumferential aneurysmal wall enhancement in VWI were relevant predictive risk factors for the development of R-ANs. We further found that repeated EVTs for R-ANs is a safe and effective treatment strategy. Multiple retreatment sessions achieved aneurysm stabilization and favorable aneurysm obliteration in 85.0% and 65.0% of patients, respectively, with a low complication rate (2.6%).

### Repeat recurrence after EVT

During the observational period of our study from 2016 to 2022, a total of 1,045 aneurysms were initially treated with EVT. Among them, 124 cases (11.9%) exhibited recurrence or regrowth that requiring retreatment. Previous studies [19, 20] have demonstrated the recanalization rates after EVT ranging from 10% to 33.6%, with retreatment rates between 4.7% and 12.3%,which are in line with our presented result. As for the re-recurrence after retreatment, although reports specifically addressing this issue remain limited [21, 22], the re-recanalization rates appears to be higher than after the initial EVT. In one large retrospective cohort study [21] including aneurysms requiring multiple EVT sessions, the initial recanalization rate was 7.6%, which increased to 48.8% (major recanalization, 25.6%) after the second EVT session. Our results are also showing the same trend, with an initial retreatment rate of 11.9% (124/1,045 aneurysms), and a re-retreatment rate of 20.0% (23/115 aneurysms). This increasing recanalization and retreatment rate after each EVT session may be due to technical and morphological challenges in achieving complete occlusion in certain aneurysms [21, 22]. Additionally, specific clinical factors may impede aneurysm thrombosis and occlusion, necessitating multiple EVT sessions. Furthermore, an unknown biological cascade induced by intra-aneurysm coil packing may contribute to aneurysm regrowth. Nevertheless, the higher incidence of recanalization in aneurysms requiring retreatment highlights the need for rigorous follow-up in such patients.

### Relevant risk factor for R-ANs development

Several clinical factors have been identified as risk factors for aneurysm recurrence following EVT, including age, size, neck width, location, packing density, and treatment method [8, 10, 17, 18, 23]. However, risk factors for repeat recurrence (i.e. R-AN development), remain poorly studied. Recently, Bae et al. [11] demonstrated that neck width, comorbid polycystic kidney disease, use of stents, and occlusion status after retreatment are independent risk factors for second recurrence. Similarly, another study [21] demonstrated aneurysm location (posterior circulation), size, and occlusion status after retreatment as independent risk factors for re-recurrence. In addition to these findings, we demonstrated that large size (> 10 mm), circumferential aneurysmal wall enhancement on VWI, are relevant and independent risk factors associated with R-AN development.

Among the clinical risk factors identified, this is the first to demonstrate that ‘circumferential wall enhancement on VWI’ are the strong predictor of the development of R-ANs. Recently, wall enhancement of aneurysms on VWI has gained attention as a novel diagnostic biomarker that can evaluate the nature of aneurysms non-invasively, prior to the treatment [8, 14, 16]. Several studies have reported that aneurysmal wall enhancement is associated with aneurysm instability, including higher risk of rupture, growth, and symptomatic presentation [8, 12–15]. Furthermore, recent study [8, 15] revealed that aneurysmal wall enhancement is also correlated with recurrence after EVT.

Although the precise biological mechanisms underlying aneurysmal wall enhancement remain to be fully elucidated, it is currently hypothesized to reflect inflammatory activity within the aneurysm wall and/or the presence of intraluminal microthrombi [13, 15, 16]. Supporting this notion, previous pathological studies using human specimens and animal models have demonstrated immune cell infiltration, endothelial disruption, and thrombus organization in unstable aneurysms [24, 25]. Moreover, a recent report [26] identified an association between aneurysmal wall enhancement and elevated levels of the systemic immune-inflammation index (SII) [27], suggesting that inflammation may be a key biological mechanism contributing to aneurysm instability, that is, the development of R-AN.

While the full biological cascade remains incompletely understood, our findings indicate that circumferential wall enhancement is a clinically significant biomarker associated with repeat recurrence following EVT. From this perspective, the presence of this finding prior to treatment may warrant consideration of more durable therapeutic strategies, such as adoption of flow diverters, to achieve long-term occlusion. And also, rigorous post-treatment follow-up is essential in these high-risk cases.

### Occlusion status in initial EVT

In addition to the above noted clinical risk factors, our analysis suggested that the degree of aneurysm occlusion at the initial EVT may influence the risk of developing refractory aneurysms (R-ANs). We observed a clear trend toward a lower incidence of R-ANs in cases that attained more favorable occlusion status, such as Raymond-Roy Class 1 or 2, at the initial procedure. These findings are consistent with previous literature [28, 29], which emphasizes the importance of achieving dense and stable aneurysm occlusion during the initial EVT to minimize the risk of long-term recurrence. Incomplete embolization status, such as residual neck or dome filling, may subject to ongoing hemodynamic stress, thereby promoting progressive regrowth and increasing the need for retreatment. Therefore, optimizing the embolization strategy at the initial EVT session, whether through the appropriate selection of coil type, the use of adjunctive devices such as stents or balloons, or implementation of novel techniques seems to be essential to ensure a durable outcome. Furthermore, the emergence of novel technologies, such as flow diverters (FD) and intrasaccular devices like the Woven EndoBridge (WEB), may further refine the relationship between initial occlusion status and long-term disabilities in future studies.

### Treatment strategy for R-ANs

Regarding the treatment strategy for R-AN, consistent with the findings of previous cohort studies, we clarified that multiple EVT sessions are safe and effective. However, the optimal treatment approach for R-AN remains controversial. While some reports suggest that clipping offers better durability and lower recurrence rates [9, 30], additional EVTs are generally preferred in actual clinical practice because of their safety and efficacy [11, 21, 22, 31]. Slob et al. [32] reported coiling of 41 aneurysms without any periprocedural complications, and Henkes et al. [19] also found no additional risk with second or even serial EVTs compared to initial treatment. Procedural rupture rates were decreased with repeated attempts (first, 3%; second, 1.1%), with no ruptures in the third or further subsequent treatment sessions [19]. In our retrospective analysis, the incidence rate of major periprocedural complications was 2.6%, with no aneurysm ruptures occurring during the treatment period.

Importantly, none of the R-AN cases in our series underwent microsurgical clipping. While clipping remains a potential curative option, its applicability may be limited in R-ANs due to technical challenges such as previously placed coils and stents, which can complicate surgical exposure and manipulation. Given these considerations, repeat EVTs would be considered a first-line treatment strategy for R-ANs, not only for its minimally invasive nature but also for its demonstrated safety and efficacy in appropriately selected cases.

## Limitation

The retrospective design, disease heterogeneity, and small sample size, particularly of the R-AN group, represent major limitations of this study. Additionally, the single-center nature of our cohort further limits the generalizability of our findings to broader clinical settings. While the small sample size of the R-AN group is acknowledged, certain predictors, such as large aneurysm size and circumferential wall enhancement on VWI, demonstrated notably high odds ratios, supporting their potential utility as clinically relevant markers of refractory aneurysms. Given that Gd-enhanced VWI can be easily implemented using standard MRI protocols, these imaging biomarkers may be applicable even in general neurosurgical settings, not limited to high-volume centers. Therefore, despite inherent limitations, the ability to identify high-risk aneurysms preoperatively has important implications for real-world treatment decision-making.

Nonetheless, to truly validate the clinical utility of these predictors and clinical applicability, large-scale, prospective, multicenter clinical trials are warranted. Such studies should incorporate standardized imaging protocols, including VWI, and establish unified follow-up regimens and treatment selection criteria. The development of such well-designed trials is essential to define optimal management strategies for refractory cerebral aneurysms.

Furthermore, this study reported the overall safety and efficacy of different EVT approaches, including simple coiling, stent-assisted coil embolization, and the more recently developed FD treatment, without stratifying the outcomes by each treatment modality. Although the proportion of treatment modalities did not differ significantly between the R-AN and control groups (as shown in Table 1), this heterogeneity remains a critical limitation when assessing the comparative efficacy of each approach. To reduce this treatment-related bias, future prospective studies with standardized treatment selection criteria and stratified analyses by modality are warranted to identify the optimal therapeutic approach.

## Conclusions

Large size, and circumferential wall enhancement in VWI are independent predictors of repeat recurrence, R-AN development. Careful follow-up after EVT should be performed, particularly in patients with these risk factors. Although the appropriate therapeutic strategy should be selected on a case-by-case basis, repeated EVT sessions were safe and efficient. Further large, well-designed clinical trials are essential to better understand R-AN.

## Data Availability

No datasets were generated or analysed during the current study.

## References

[1] Toyoda K, Yoshimura S, Nakai M, Koga M, Sasahara Y, Sonoda K et al (2022) Twenty-year change in severity and outcome of ischemic and hemorrhagic strokes. JAMA Neurol 79(1):61–69. 10.1001/jamaneurol.2021.4346 34870689 10.1001/jamaneurol.2021.4346 PMC8649912

[2] Algra AM, Lindgren A, Vergouwen MDI, Greving JP, van der Schaaf IC, van Doormaal TPC et al (2019) Procedural clinical complications, case-fatality risks, and risk factors in endovascular and neurosurgical treatment of unruptured intracranial aneurysms: a systematic review and meta-analysis. JAMA Neurol 76(3):282–293. 10.1001/jamaneurol.2018.4165 30592482 10.1001/jamaneurol.2018.4165 PMC6439725

[3] Haverkamp C, Kaier K, Shah M, von Zur MC, Beck J, Urbach H et al (2024) Cerebral aneurysms: Germany-wide real-world outcome data of endovascular or neurosurgical treatment from 2007 to 2019. J Neurointerv Surg 16(4):365–371. 10.1136/jnis-2023-020181 37290919 10.1136/jnis-2023-020181 PMC10958314

[4] Imamura H, Sakai N, Sakai C, Fujinaka T, Ishii A, Investigators J-N (2014) Endovascular treatment of aneurysmal subarachnoid hemorrhage in Japanese registry of neuroendovascular therapy (JR-NET) 1 and 2. Neurol Med Chir (Tokyo) 54(2):81–90. 10.2176/nmc.oa.2013-0228 24390181 10.2176/nmc.oa.2013-0228 PMC4508712

[5] Molyneux A, Kerr R, Stratton I, Sandercock P, Clarke M, Shrimpton J et al (2002) International subarachnoid aneurysm trial (ISAT) of neurosurgical clipping versus endovascular coiling in 2143 patients with ruptured intracranial aneurysms: a randomised trial. Lancet 360(9342):1267–1274. 10.1016/s0140-6736(02)11314-6 12414200 10.1016/s0140-6736(02)11314-6

[6] Molyneux AJ, Birks J, Clarke A, Sneade M, Kerr RS (2015) The durability of endovascular coiling versus neurosurgical clipping of ruptured cerebral aneurysms: 18 year follow-up of the UK cohort of the international subarachnoid aneurysm trial (ISAT). Lancet 385(9969):691–697. 10.1016/S0140-6736(14)60975-2 25465111 10.1016/S0140-6736(14)60975-2 PMC4356153

[7] Spetzler RF, McDougall CG, Zabramski JM, Albuquerque FC, Hills NK, Russin JJ et al (2015) The barrow ruptured aneurysm trial: 6-year results. J Neurosurg 123(3):609–617. 10.3171/2014.9.JNS141749 26115467 10.3171/2014.9.JNS141749

[8] Hara T, Matsushige T, Yoshiyama M, Hashimoto Y, Kobayashi S, Sakamoto S (2023) Association of circumferential aneurysm wall enhancement with recurrence after coiling of unruptured intracranial aneurysms: a preliminary vessel wall imaging study. J Neurosurg 138(1):147–153. 10.3171/2022.4.JNS22421 35594885 10.3171/2022.4.JNS22421

[9] Sweid A, El Naamani K, Abbas R, Starke RM, Badih K, El Hajjar R et al (2022) Clipping could be the best treatment modality for recurring anterior communicating artery aneurysms treated endovascularly. Neurosurgery 90(5):627–635. 10.1227/neu.0000000000001905 35285450 10.1227/neu.0000000000001905 PMC9514745

[10] Crobeddu E, Lanzino G, Kallmes DF, Cloft HJ (2013) Review of 2 decades of aneurysm-recurrence literature, part 1: reducing recurrence after endovascular coiling. AJNR Am J Neuroradiol 34(2):266–270. 10.3174/ajnr.A3032 22422180 10.3174/ajnr.A3032 PMC7965107

[11] Bae JW, Oh HS, Hong CE, Kim KM, Yoo DH, Kang HS et al (2024) Extended monitoring of re-coiled cerebral aneurysms after initial postcoiling recanalization: safety and durability of repeat coil embolization. J Neuroradiol 51(1):59–65. 10.1016/j.neurad.2023.05.006 37247754 10.1016/j.neurad.2023.05.006

[12] Omodaka S, Endo H, Niizuma K, Fujimura M, Endo T, Sato K et al (2018) Circumferential wall enhancement on magnetic resonance imaging is useful to identify rupture site in patients with multiple cerebral aneurysms. Neurosurgery 82(5):638–644. 10.1093/neuros/nyx267 28586440 10.1093/neuros/nyx267

[13] Omodaka S, Endo H, Niizuma K, Fujimura M, Inoue T, Endo T et al (2018) Circumferential wall enhancement in evolving intracranial aneurysms on magnetic resonance vessel wall imaging. J Neurosurg 131(4):1262–1268. 10.3171/2018.5.JNS18322 30485237 10.3171/2018.5.JNS18322

[14] Omodaka S, Endo H, Niizuma K, Endo T, Sato K, Saito A et al (2022) Wall enhancement in unruptured posterior communicating aneurysms with oculomotor nerve palsy on magnetic resonance vessel wall imaging. J Neurosurg. 137(3):668–674. 10.3171/2021.11.JNS212249. (**1-7**) 35061982 10.3171/2021.11.JNS212249

[15] Hashimoto Y, Matsushige T, Kawano R, Hara T, Kobayashi S, Kaneko M et al (2024) High signal intensity of the intraaneurysmal sac on T1 CUBE imaging as a predictor of aneurysm stability after coil embolization. J Neurosurg 140(1):144–152. 10.3171/2023.5.JNS23616 37439478 10.3171/2023.5.JNS23616

[16] Endo H, Mori N, Mugikura S, Niizuma K, Omodaka S, Takase K et al (2022) Quantitative assessment of microstructural evolution of intracranial aneurysm wall by vessel wall imaging. Neuroradiology 64(7):1343–1350. 10.1007/s00234-021-02877-7 34997283 10.1007/s00234-021-02877-7

[17] Roy D, Milot G, Raymond J (2001) Endovascular treatment of unruptured aneurysms. Stroke 32(9):1998–2004. 10.1161/hs0901.095600 11546888 10.1161/hs0901.095600

[18] Raymond J, Guilbert F, Weill A, Georganos SA, Juravsky L, Lambert A et al (2003) Long-term angiographic recurrences after selective endovascular treatment of aneurysms with detachable coils. Stroke 34(6):1398–1403. 10.1161/01.STR.0000073841.88563.E9 12775880 10.1161/01.STR.0000073841.88563.E9

[19] Henkes H, Fischer S, Liebig T, Weber W, Reinartz J, Miloslavski E et al (2006) Repeated endovascular coil occlusion in 350 of 2759 intracranial aneurysms: safety and effectiveness aspects. Neurosurgery 58(2):224–32. 10.1227/01.NEU.0000194831.54183.3F. (**discussion -32**) 16462475 10.1227/01.NEU.0000194831.54183.3F

[20] Ries T, Siemonsen S, Thomalla G, Grzyska U, Zeumer H, Fiehler J (2007) Long-term follow-up of cerebral aneurysms after endovascular therapy prediction and outcome of retreatment. AJNR Am J Neuroradiol 28(9):1755–1761. 10.3174/ajnr.A0649 17885238 10.3174/ajnr.A0649 PMC8134224

[21] Lee J, Lim JW, Cho YD (2018) Follow-up outcomes after re-embolization for recanalized aneurysms after initial coiling: further recurrence rates and related risk factors. World Neurosurg 114:e508–e517. 10.1016/j.wneu.2018.03.017 29530696 10.1016/j.wneu.2018.03.017

[22] Matsukawa H, Tanikawa R, Kamiyama H, Noda K, Uchida K, Shirakawa M et al (2021) Outcome of retreatment for recurrent saccular cerebral aneurysms: a propensity score-matched analysis. Neurosurg Rev 44(2):935–944. 10.1007/s10143-020-01259-6 32086690 10.1007/s10143-020-01259-6

[23] Chalouhi N, Jabbour P, Singhal S, Drueding R, Starke RM, Dalyai RT et al (2013) Stent-assisted coiling of intracranial aneurysms: predictors of complications, recanalization, and outcome in 508 cases. Stroke 44(5):1348–1353. 10.1161/STROKEAHA.111.000641 23512976 10.1161/STROKEAHA.111.000641

[24] Hokari M, Nakayama N, Nishihara H, Houkin K (2015) Pathological findings of saccular cerebral aneurysms-impact of subintimal fibrin deposition on aneurysm rupture. Neurosurg Rev 38(3):531–40. 10.1007/s10143-015-0628-0. (**discussion 40**) 25860660 10.1007/s10143-015-0628-0

[25] Shimoda Y, Nakayama N, Moriwaki T, Abumiya T, Kawabori M, Kurisu K et al (2022) Induction of large cerebral aneurysms by intraperitoneal administration of beta-aminopropionitrile fumarate in male rats. J Neurosurg Sci 66(3):220–227. 10.23736/S0390-5616.19.04819-7 32031355 10.23736/S0390-5616.19.04819-7

[26] Peng F, Xia J, Niu H, Feng X, Zheng T, He X et al (2023) Systemic immune-inflammation index is associated with aneurysmal wall enhancement in unruptured intracranial fusiform aneurysms. Front Immunol 14:1106459. 10.3389/fimmu.2023.1106459 36776878 10.3389/fimmu.2023.1106459 PMC9911448

[27] Kurisu K, Osanai T, Morishima Y, Ito M, Uchino H, Sugiyama T et al (2024) Systemic immune-inflammation index in dural arteriovenous fistula: a feasible biomarker reflecting its clinical characteristics. Acta Neurochir (Wien) 166(1):180. 10.1007/s00701-024-06075-1 38627314 10.1007/s00701-024-06075-1

[28] Chalouhi N, Starke RM, Koltz MT, Jabbour PM, Tjoumakaris SI, Dumont AS et al (2013) Stent-assisted coiling versus balloon remodeling of wide-neck aneurysms: comparison of angiographic outcomes. AJNR Am J Neuroradiol 34(10):1987–1992. 10.3174/ajnr.A3538 23639562 10.3174/ajnr.A3538 PMC7965428

[29] Tosello RT, Batista UC, Pereira BJA, Piske RL (2017) Packing density necessary to reach a high complete occlusion rate in circumferential unruptured intracranial aneurysms treated with stent-assisted coil embolization. AJNR Am J Neuroradiol 38(10):1973–1977. 10.3174/ajnr.A5303 28751517 10.3174/ajnr.A5303 PMC7963617

[30] Lejeune JP, Karnoub MA, Devalckeneer A, Bretzner M, Bourgeois P, Aboukais R (2024) Microsurgical management of recurrent intracranial aneurysm after endovascular treatment: a series of 60 consecutive patients. J Neurosurg 141(5):1235–1243. 10.3171/2024.3.JNS24116. (**1-9**) 38941640 10.3171/2024.3.JNS24116

[31] Hokari M, Kazumara K, Nakayama N, Ushikoshi S, Sugiyama T, Asaoka K et al (2016) Treatment of recurrent intracranial aneurysms after clipping: a report of 23 cases and a review of the literature. World Neurosurg 92:434–444. 10.1016/j.wneu.2016.05.053 27241096 10.1016/j.wneu.2016.05.053

[32] Slob MJ, Sluzewski M, van Rooij WJ, Roks G, Rinkel GJ (2004) Additional coiling of previously coiled cerebral aneurysms: clinical and angiographic results. AJNR Am J Neuroradiol 25(8):1373–1376 15466335 PMC7975452

